# A comparison of time dependent Cox regression, pooled logistic regression and cross sectional pooling with simulations and an application to the Framingham Heart Study

**DOI:** 10.1186/s12874-016-0248-6

**Published:** 2016-11-03

**Authors:** Julius S. Ngwa, Howard J. Cabral, Debbie M. Cheng, Michael J. Pencina, David R. Gagnon, Michael P. LaValley, L. Adrienne Cupples

**Affiliations:** 1 Department of Biostatistics, Boston University, School of Public Health, 801 Massachusetts Ave, CT 3rd Floor, Boston, MA 02118 USA; 2 Department of Biostatistics, Johns Hopkins Bloomberg School of Public Health, 615 North Wolfe St, Baltimore, MD 21205 USA; 3 Department of Biostatistics and Bioinformatics, Duke University, School of Medicine, 2400 Pratt St, 7021 North Pavilion, Durham, NC 27705 USA; 4 National Heart, Lung, and Blood Institute’s Framingham Heart Study, Framingham, MA 01702 USA

**Keywords:** Time dependent covariate model (TDCM), Cross sectional pooling (CSP), Pooled logistic regression (PLR), Longitudinal and survival data

## Abstract

**Background:**

Typical survival studies follow individuals to an event and measure explanatory variables for that event, sometimes repeatedly over the course of follow up. The Cox regression model has been used widely in the analyses of time to diagnosis or death from disease. The associations between the survival outcome and time dependent measures may be biased unless they are modeled appropriately.

**Methods:**

In this paper we explore the Time Dependent Cox Regression Model (TDCM), which quantifies the effect of repeated measures of covariates in the analysis of time to event data. This model is commonly used in biomedical research but sometimes does not explicitly adjust for the times at which time dependent explanatory variables are measured. This approach can yield different estimates of association compared to a model that adjusts for these times. In order to address the question of how different these estimates are from a statistical perspective, we compare the TDCM to Pooled Logistic Regression (PLR) and Cross Sectional Pooling (CSP), considering models that adjust and do not adjust for time in PLR and CSP.

**Results:**

In a series of simulations we found that time adjusted CSP provided identical results to the TDCM while the PLR showed larger parameter estimates compared to the time adjusted CSP and the TDCM in scenarios with high event rates. We also observed upwardly biased estimates in the unadjusted CSP and unadjusted PLR methods. The time adjusted PLR had a positive bias in the time dependent Age effect with reduced bias when the event rate is low. The PLR methods showed a negative bias in the Sex effect, a subject level covariate, when compared to the other methods. The Cox models yielded reliable estimates for the Sex effect in all scenarios considered.

**Conclusions:**

We conclude that survival analyses that explicitly account in the statistical model for the times at which time dependent covariates are measured provide more reliable estimates compared to unadjusted analyses. We present results from the Framingham Heart Study in which lipid measurements and myocardial infarction data events were collected over a period of 26 years.

**Electronic supplementary material:**

The online version of this article (doi:10.1186/s12874-016-0248-6) contains supplementary material, which is available to authorized users.

## Background

A time dependent explanatory variable is one whose value for a subject may change over the period of time that the subject is observed [1, 2]. The most common type of time dependent covariate is a repeated measurement on a subject or perhaps a change in the subject’s treatment. Data for a subject can be presented as multiple observations, each of which applies to a time interval of observation, which is usually the time period between the exams when the longitudinal measures were recorded. The Cox proportional hazard regression model is often used to analyze covariate information that changes over time, with the hazard proportional to the instantaneous probability of an event at a particular time [3, 4]. Typical settings where time dependent covariates occur include HIV studies in which baseline characteristics are recorded and immunological measures such as CD4+ lymphocyte counts or viral load are measured repeatedly to assess patients’ health until HIV conversion. Therneau and Grambsch considered a well-known example of the time dependent Cox model (TDCM) using the Stanford Heart Transplant Program [3].

There is extensive literature and a wide range of statistical packages for modeling time dependent covariate data. Some previous work includes Fisher and Lin [1]; Cupples et al. [5]; D’Agostino et al. [6]; Pepe and Cai [7]; Prentice and Gloeckler [8]; Abbott [9]; Green and Symons [10]; Ingram and Kleinman [11]; Kalbfleisch and Prentice [12]; Wu and Ware [13].

Models that can accommodate time-dependent covariates are commonly used in biomedical research but sometimes do not explicitly adjust for time in the model. Not adjusting for time can yield different estimates of association compared to a model that adjusts for time. In order to address the question of how different these estimates are, we compare three methods that model the association between a longitudinal process and a time-to-event outcome. We consider the TDCM in which the longitudinal measures are used as time dependent covariates in a Cox model [4]. We compare the TDCM to Pooled Logistic Regression (PLR) and Cross Sectional Pooling (CSP). The PLR and CSP methods pool observations over disjoint time intervals of equal length into a single sample in order to predict the short term risk of the event. The CSP, unlike the PLR, utilizes information on the length of time to event in each interval as well as whether or not the event occurs. We consider models that adjust and do not adjust for the timing of the longitudinal measures in the PLR and also time-interval and non-time-interval models for the CSP. In this paper we refer to all CSP stratified models with time intervals as time adjusted models.

The Framingham Heart Study (FHS) has been collecting data prospectively since 1948 to examine the relationship of potential risk factors to the development of cardiovascular disease [5]. Since risk factors for disease have been collected prospectively and repeatedly measured over time (every 2–4 years), the FHS provides an important example to study various approaches for survival analysis with repeated measures. The PLR has been frequently employed in the analysis of FHS data [5, 6]. For FHS, the PLR treats the two to four-year examination interval as a mini-follow up study in which the current risk factors are updated at the interval start to predict events during the interval. In this paper we applied the above methods to FHS data in which triglycerides (TG) were measured at generally comparable time intervals, about every 4 years, over a 26-year period in the FHS Offspring cohort. Time to myocardial infarction was also recorded for each participant, with some subjects remaining free of myocardial infarction at the end of the study period and these subjects were administratively censored at that time. The research protocols of the Framingham Heart Study are reviewed annually by the Institutional Review Board of the Boston University Medical Center and by the Observational Studies Monitoring Board of the National Heart, Lung and Blood Institute. Participants signed a consent form approved by the Institutional Review Board.

Our main objectives are to: (i) compare the TDCM to Pooled Logistic Regression (time adjusted and unadjusted models) and Cross Sectional Pooling (time adjusted and unadjusted models) in simulation studies; and (ii) illustrate the methods and compare their results by applying them to FHS data. We begin by presenting an overview of these methods for modeling time dependent covariates in the context of longitudinal and survival data. We then evaluate the methods using simulation studies and conclude with a discussion of the results from the simulation and the Framingham Heart Study.

## Methods

In modeling longitudinal and survival data the main focus may be on the longitudinal component, the survival component, or both, depending on the objectives of a study. When the focus is on one aspect, the other component is then secondary; so its parameters may be viewed as nuisance parameters [14]. Our goal is to characterize the relation between time-to-event (dependent) and the longitudinal measures (independent) in models that account for the time at which the longitudinal measures are recorded.

In this section, we consider methods for modeling the association between longitudinal measures and time-to-event data in a survival model. We consider the underlying model to include longitudinal response data and time-to-event data for a sample of size n, consisting of [*T*_i_^*^, *δ*_*i*_, [*Y*_*i*_(*t*), 0 ≤ *t* ≤ *T*_*i*_], *i* = 1, 2, …, *n*] observations where *T*_*i*_ is the time-to-event for the *i* ^*th*^ subject. The vector *Y*_*i*_(*t*) = [*Y*_*i*1_(*t*), …, *Y*_*ip*_(*t*)]^*t*^ is a set of longitudinal measures, and *m*_*i*_ ≤ *p* is the number of time intervals for the *i* ^*th*^ subject. In addition, each subject has possibly right censored failure *T*_*i*_ = min(*T*_i_* , *C*_*i*_) and the event indicator *δ*_*i*_ (*δ*_*i*_ = 1 *if T*_i_* ≤ *C*_*i*_; *δ*_*i*_ = 0 *if T*_i_* > *C*_*i*_). The parameter *δ*_*i*_ indicates whether the observed failure time is a true failure time *T*_i_*, or a censoring time *C*_*i*_.

The study design that we consider in our paper has fixed time points where each person has observations at which covariates are measured. Such a study design is common for longitudinal studies. In particular, individuals are measured for a time-varying covariate (*Y*) at the beginning of each time interval and all intervals are of the same length (in our simulations, 5 years). In this context, the regression coefficients represent the association between Y and an event that occurs during the subsequent interval. We require this study design in order to compare the PLR model, which does not explicitly consider time, with those approaches that do incorporate time into the model. For CSP and TDCM, time intervals of equal length are not needed as an assumption of the model. In all the models that we considered, the assumption is that the time dependent covariates remain constant between examination times.

### Time dependent Cox regression modeling

A time dependent explanatory variable is one that may change over the period of time that the subject is observed [2]. The most common time dependent covariates are repeated measures on a subject or a change in the subject’s treatment. A proportional hazard model is often used to analyze covariate information that changes over time. One way of handling time-dependent repeated measurements in SAS is to specify programming statements to capture the appropriate covariate values of the subjects in each time interval of observation. TDCM can be fit using the standard partial likelihood for the Cox model where the values for the time dependent covariates are updated in each of the event-specific likelihood terms.

The hazard for the TDCM at time *t* can be written as:

1 $$ h\left(t;\ {Y}_i,\ {X}_i\right)={h}_0(t)* exp\left({Y}_i{(t)}^T\beta + {X_i}^T\alpha \right) = {h}_0(t)* exp\left({\displaystyle \sum_{k=1}^{m_i}}{Y}_{ik}{(t)}^T{\beta}_k + {X_i}^T\alpha \right) $$

Where *h*_0_(*t*) represents the baseline hazard function, *X*_*i*_ is a vector of time invariant explanatory covariates with regression parameters. *Y*_*ik*_(*t*) is a general covariate form in which *m*_*i*_ = *p* is the number of longitudinal measures for each subject *i*. We define *t*_1_ < *t*_2_ < *t*_3_ < … < *t*_*D*_ as a set of ordered observed event times with *D* unique failure times and *Y*_*i*_(*t*_*i*_) as the covariates associated with the individual whose failure time is *t*_*i*_ for *i* = 1, …, *D* failure times. The parameter *β*_*k*_ measures the association between the observed longitudinal measures and the hazard of failure time *h*(*t*)*.* The risk set *R*(*t*_*i*_) at failure time *t*_*i*_ is the set of all individuals who are still under study at a time just prior to *t*_*i*_. In most applications, *β*_*k*_ = 0 for intervals other than the current one (*t*_*i*_). The partial likelihood based on the hazard function specified in (1) can be written as:

2 $$ L\left(\alpha,\ \beta \right)={\displaystyle \prod_{i=1}^D}\left\{\frac{exp\left({Y}_i{\left({t}_i\right)}^T\beta + {X_i}^T\alpha \right)}{{\displaystyle {\sum}_{l\epsilon R\left({t}_i\right)}} exp\left[{Y}_l{\left({t}_i\right)}^T\beta + {X_l}^T\alpha \right]}\right\} $$

In the partial likelihood above, each term is the conditional probability of choosing individual *i* to fail from the risk set, given the risk set at failure time *t*_*i*_ and given that one failure occurs. The inference is similar to the Cox model. The only difference is that the values of *Y*_*i*_(*t*_*i*_) now change for each risk set. The *α* and *β* estimates can be obtained by maximizing the likelihood in (2). In TDCM the covariates are measured repeatedly and an assumption of this model is that the hazard depends on the covariate through its current value.

### Pooled repeated observations

The use of standard logistic regression techniques to estimate hazard rates was detailed by Efron [15]. His approach, known as partial logistic regression, entailed the use of parametric logistic regression modeling on censored data to obtain estimates and standard errors. The pooled repeated observations approach, described by Cupples et al. [5], has been frequently employed in the Framingham Heart Study. In this method each observation interval is considered a mini-follow up study in which the current risk factors are updated to predict events in the interval. Once an individual has an event in a particular interval all subsequent intervals from that individual are excluded from the analysis.

#### Pooled logistic regression (PLR)

In PLR, logistic regression is used to link predictors to the event outcome. The outcome is an event indicator, which records whether an event occurs in the interval or not and does not account for when the event occurs within the interval. A response occurring near the beginning of a follow-up period is treated the same in analysis as one occurring at the end of that period. This model relates the probability of an event occurring in an interval to a logistic function of the risk factors [5].

3 $$ Ln\ \left(\frac{P\left({t}_k,{Y}_i,\ {X}_i\right)}{1-P\left({t}_k,{Y}_i,\ {X}_i\right)}\right)={\beta}_o + {Y}_i{\left({t}_k\right)}^T\boldsymbol{\gamma} +{X_i}^T\alpha +{\theta}_k $$

The parameter *β*_*o*_ is the intercept for the logistic model. The *Y*_*i*_(*t*_*k*_) represent the observed longitudinal measures for the interval; the parameter *θ*_*k*_ denotes the effect of time *t*_*k*_. The time point *t*_*k*_ is an element of the vector representing when the longitudinal measures were recorded. Thus, this model adjusts for the interval in which the observations were made. In our application of this model, we assumed a linear trend on the time effects *θ*_*k*_. One drawback of PLR is that the model does not utilize information for the point in time during the interval at which an event occurs or the exact time in an interval that an individual is lost to follow-up. Thus, the contribution of the risk factor to disease is dependent on the length of follow-up period [10]. While there may be concern with the PLR regarding the dependence of multiple records within an individual contributing to several intervals, Allison (2010) has noted that in working with a dataset with multiple records for intervals within each individual there is no inflation of test statistics resulting from a lack of independence [16]. This property is due to the fact that the likelihood factors into a distinct term for each interval. Allison also cautioned that this conclusion may not apply when the dataset includes multiple events for each individual. Singer and Willett (2003) also noted that the hazard, or odds in PLR, describes the conditional probability of event occurrence, where the conditioning depends upon the individual survival until that particular time period. This allows all records within the person-period dataset to be considered as conditionally independent [17].

We should also note that the PLR model provides estimates of conditional odds ratios for having the event in an interval rather than of the hazard ratio. Efron [15] discussed the use of the logistic model for survival data and showed that this approach gives direct estimates of the hazard rate and provides approximate standard errors. They refer to this parametric model as partial logistic regressions due to its connection to Cox’s (1975, ex. 2) theory of partial likelihood. Moreover, Efron’s conditional logistic regression model and pooled logistic regression are equivalent when the length of time interval tends towards zero. Green and Symons [10] found that when the follow-up period is short and the event is rare, the logistic regression estimates and their standard errors approximate those from the proportional hazards mode.

#### Cross sectional pooling (CSP)

The CSP uses Cox regression within interval to utilize information on the length of time to event within each interval as well as whether or not the event occurs. The model relates the instantaneous risk of an event to the longitudinal measures though a hazard function.

4 $$ {h}_j\left(t;{Y}_i,\ {X}_i\right)={h}_{j0}(t)* \exp \left({Y}_i{(t)}^T\boldsymbol{\gamma} +{X_i}^T\alpha \right) $$

Where *h*_*j*0_(*t*) represents the baseline hazard function for the *j* ^*th*^ interval, ***γ*** is the association parameter; *t* is the time-to-event in the interval. In the time adjusted CSP a stratified Cox model is implemented with time intervals (*j*) when longitudinal measurements were taken. In the unadjusted model, the hazard is assumed to be the same across all time intervals and analysis is performed without stratification. In stratified Cox with time intervals, the regression coefficients are assumed to be the same in each interval; however, the baseline hazard function may vary.

## Simulation studies

We conducted a series of simulations to evaluate the performance of the CSP, TDCM and PLR methods for modeling longitudinal and survival data. We structured the simulated data to resemble observed data from the Framingham Heart Study as the covariates were measured at specific time intervals (each ~4 years) and held fixed until the next measurement time point. For the simulations we used 5 year intervals. In Table 1, we provide the simulation model and the parameters used for simulating the longitudinal and survival data. The longitudinal trajectories were generated from a linear model with age of the participant at entry into the study as a predictor, while survival times were generated using a Weibull model to depend on the longitudinal measures and an additional set of covariates, possibly time-varying. In each 5-year time period if the survival time was less than or equal to the time period of the mini follow-up defined by the timing of longitudinal measures, then the event was considered to be observed and the time-to-event in that interval was the survival time; otherwise the time-to-event for the interval was censored at the end of the interval [18]. We assumed random non-informative right censoring for subjects remaining event free through the last time interval. We present an algorithm below for generating the simulated data.

**Table 1:** Model and parameters in simulation study

Longitudinal model			*Y*_*ij*_ = *U*_*i*1_ + *U*_*i*2_ * *t*_*ij*_ + *τ* * *Age* + *ε*_*ij*_		
Survival model			*h*(*t*) = *λ*(*t*)*exp*{*α*_1_ *Age* + *α*_2_ *Sex* + *γY*_*ij*_}		
Covariance matrix for random effects (*U*_*i*1_ , *U*_*i*2_)			$$ G = \left[\begin{array}{cc}\hfill 0.29\hfill & \hfill -0.00465\hfill \\ {}\hfill -0.00465\hfill & \hfill 0.000320\hfill \end{array}\right] $$		
# of exams	Random effects (*U*_*i*1_ , *U*_*i*2_)	Residual error (σ^2^)	Age (*α*_1_)	Sex (*α*_2_)	Link (*γ*)
6	(4.250, 0.250)	0.116	0.050	−0.500	(0.000, 0.500, 1.000)

*Y*_*ij*_: Observed Longitudinal Measures; *λ*(*t*): Baseline Hazard Function; *h*(*t*): Hazard Function

We simulated independent multivariate datasets consisting of longitudinal measures and time-to-event outcomes. The following algorithm was implemented to generate the longitudinal data using steps 1–5 and the survival data using steps 6 and 7:

Longitudinal component

1. Generate baseline covariates similar to FHS.
  - Baseline Age ~ *normal* (35,5) and Sex ~ *Bernoulli* (0.54)
2. Generate longitudinal trajectories (*φ*_*β*_(*t*_*ij*_)) for each subject (*i* = 1, 2, …, *n*) and for each time point (*j* = 1, 2, …, *m*_*i*_) using the linear model: *φ*_*β*_(*t*_*ij*_) = *U*_*i*1_ + *U*_*i*2_ * *t*_*ij*_ + *τ* * *Age*
  - Parameter estimates for the mean and variance-covariance matrix (*G*) of the random effects, covariance matrix and residual errors were obtained by fitting a random effects model to the FHS data.
  - Generate random effects (*U*_*i*1_ , *U*_*i*2_) from a bivariate normal distribution with mean and variance-covariance (*G*) obtained (2a). The random effects (*U*_*i*1_ , *U*_*i*2_) represent the intercept and slope.
3. Generate the observed longitudinal measures (*Y*) from a multivariate normal distribution with mean *φ*_*β*_(*t*_*ij*_) and variance (*V*): $$ V={Z}_iG{Z_i}^T+{R}_i,\kern0.5em  where\ {Z}_i = \left[\begin{array}{rr}\hfill 1& \hfill 0\\ {}\hfill 1& \hfill 5\\ {}\hfill 1& \hfill 10\\ {}\hfill 1& \hfill 15\\ {}\hfill 1& \hfill 20\\ {}\hfill 1& \hfill 25\end{array}\right]\  and\ {R}_i \sim diagmatrix\left({\upsigma}^2\right) $$

In our simulation models the continuously changing values of the triglycerides (covariates) are measured at regular 5 year intervals. The models are designed to capture the covariate measurements at specific longitudinal time points similar to the Framingham Heart Study.

Survival Component:

4. Choose parameter estimate values for Age, Sex and the link parameter which measures the strength of the association between the longitudinal measures and the time-to-event.
5. Generate the time-to-event (*T*) for each time period in which the longitudinal measures were taken, from the inverse of the cumulative hazard distribution. Survival times generated with the Cox proportional hazard model using the Exponential and Weibull distributions. When the shape is equal to 1, the Weibull distribution equals the exponential distribution. By varying the shape parameter and the scale parameter, the required event rates (10 %, 50 %, and 90 %) can be attained for the survival data.

*h*(*t*; *Age*, *Sex*, *Y*_*ij*_) = *λ*(*t*)*exp*{*α*_1_ *Age* + *α*_2_ *Sex* + ***γ*** *Y*_*ij*_}.

Survival times are generated for each interval to depend on the longitudinal measures at the beginning of the interval and a set of covariates (Age at each exam and Sex). The survival time for each participant was computed by considering the cumulative survival time across intervals until an event occurred. Subjects without an event at the last interval are censored after the 5-year period of the interval.

The parameter estimates used in our simulations for the random effects, covariance matrix and residual errors were obtained by fitting a random effects model to the FHS Data. Baseline age at entry to the study was simulated from a normal distribution with mean 35 and standard deviation 5; sex was assigned according to a draw from a Bernoulli distribution with proportion female = 0.54; these parameters are similar to those of the FHS Data. The observed longitudinal measures (*Y*_*ij*_) were generated from a multivariate normal distribution with means and variances specified above in the simulation scheme. Survival time was generated for each interval using the value of age from the start of the interval. The survival time for each participant was computed by considering the cumulative survival time across intervals until an event occurred. Each replicated data set was simulated to contain 1000 subjects with up to 6 observation intervals. We fit the methods described in section 2 to analyze each of 1000 replicated data sets and used 10,000 replicates to evaluate Type I error. We assessed the performance of these methods using bias, accuracy and coverage. Bias was assessed as the deviation in the estimate from the true simulated parameter. Mean square error (MSE) provided a measure of overall accuracy by incorporating the bias and the variability. Coverage of the confidence interval was the proportion of times the obtained confidence interval contains the true specified value.

We implemented the following methods for analysis: (1) Unadjusted Cross-sectional pooling (CSP_UN); (2) Adjusted Cross-sectional pooling (CSP_AD); (3) Time dependent Cox regression models (TDCM); (4) Unadjusted Pooled logistic regression (PLR_UN); (5) Adjusted Pooled logistic regression (PLR_AD). In Table 2 we provide a comparison of the similarities and differences across the different methods. In the CSP and PLR methods Age at each exam was included in the model as a time varying covariate; the data structure had multiple rows per subject where each row was considered a mini-follow up study in which the current risk factors were updated to predict events in the interval. In the TDCM the baseline Age variable was included in the model; the data structure was a single row per subject where the overall survival time was specified for each subject for analysis in SAS. We also ran a model in which a time dependent Age was implemented for the TDCM and we obtained the same results. For CSP and PLR the Age at each exam was calculated by adding the difference in time from the current exam and the first exam to the Age at baseline. In the analysis with these methods, updating age did not make a difference as any update occurred by the same amount for everyone. In the time adjusted PLR, we adjusted for the time interval in which the longitudinal measures were recorded by including a time variable (coded as 0, 5, 10, 15, 20 and 25) in the model. In the time adjusted CSP we used a stratified Cox with time intervals to adjust for the different time intervals in which the longitudinal measures were recorded. The statistical analyses were performed using SAS Software (version 9.3; SAS Institute, Cary, NC) and data simulation was performed in R (R Development Core Team, 2012).

**Table 2:** Summary of methods

Characteristics	CSP_UN	CSP_AD	TDCM	PLR_UN	PLR_AD
Rows per subject	Multiple	Multiple	Single	Multiple	Multiple
Regression model	Cox	Stratified Cox	Cox	Logistic	Time adjusted logistic
Outcome	Time-to-event	Time-to-event	Time-to-event	Binary	Binary
Censoring in interval permitted	Yes	Yes	Yes	No	No
Time adjusted	No	Yes	Yes	No	Yes
Age covariate	Time varying	Time varying	Fixed	Time varying	Time varying
Sex covariate	Fixed	Fixed	Fixed	Fixed	Fixed
Estimate (ratio)	Hazard	Hazard	Hazard	Odds	Odds

*Abbreviations*: *CSP_UN* Unadjusted Cross Sectional Pooling, *CSP_AD* Adjusted Cross Sectional Pooling, *PLR_UN* Unadjusted Pooled Logistic Regression, *PLR_AD* Adjusted Pooled Logistic Regression, *TDCM* Time Dependent Cox Regression Modeling

## Results

We present results from the simulation studies for the methods described in section 2. Type I errors were computed for the longitudinal effect on survival, (γ = 0), using 10,000 replicates and a sample size of 1000 (Table 3). In all simulation schemes the time adjusted CSP (CSP_AD) and TDCM provided identical results, as expected. The time adjusted and the unadjusted methods provided Type I error rates close to the nominal level of 0.05, with all results less than or equal to 10 % deviation from the nominal levels.

**Table 3:** Type I error for longitudinal effect on survival

*N* = 1000, γ = 0^a^				
Event rate	CSP_UN	CSP_AD & TDCM	PLR_UN	PLR_AD
90 %	0.048	0.047	0.048	0.048
50 %	0.054	0.055	0.053	0.054
10 %	0.048	0.048	0.048	0.048

^a^Type I Error rate based on 10,000 simulations

*Abbreviations*: *CSP_UN* Unadjusted Cross Sectional Pooling, *CSP_AD* Adjusted Cross Sectional Pooling, *PLR_UN* Unadjusted Pooled Logistic Regression, *PLR_AD* Adjusted Pooled Logistic Regression, *TDCM* Time Dependent Cox Regression Modeling

**Fig. 1:**
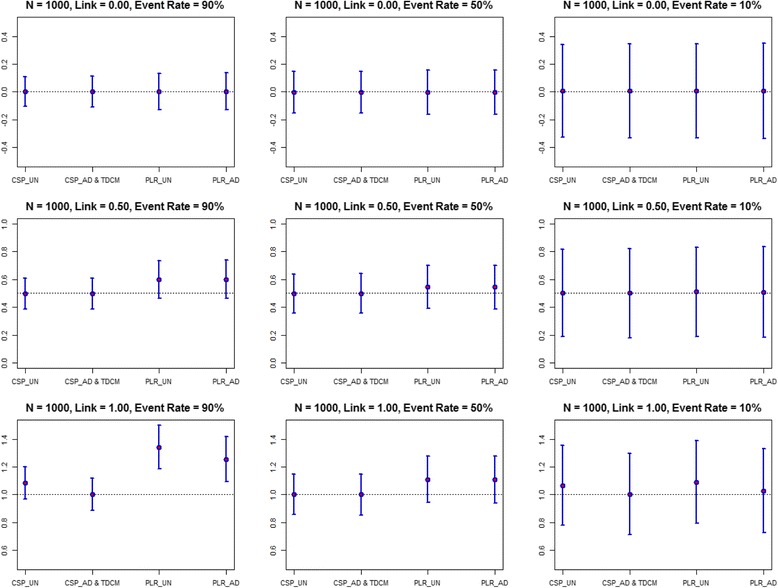
Estimates and Confidence Intervals for Association Parameter (*N* = 1000). Values are presented as estimates and 95 % confidence intervals for the link parameter. Varying link parameter (0.00, 0.50, and 1.00); varying event rates (10 %, 50 %, and 90 %). Abbreviations: CSP_UN: Unadjusted Cross Sectional Pooling; CSP_AD: Adjusted Cross Sectional Pooling; PLR_UN: Unadjusted Pooled Logistic Regression; PLR_AD: Adjusted Pooled Logistic Regression; TDCM: Time Dependent Cox Regression Modeling

We varied the event rate (90 %, 50 % and 10 %) and the association parameter (γ = 0.00, 0.50, and 1.00). The Age (*α*_1_ = 0.050) and Sex (*α*_2_ = − 0.500) parameters were constant in all the models. In Table 4 and Fig. 1 we present the estimates, SEs, coverage probability, bias and MSE for the longitudinal effect on survival using the time dependent Age simulation scheme. The TDCM and the time adjusted CSP (identical results) showed lower bias and higher coverage probability compared to the other methods. The PLR methods provided higher estimates compared to the other methods with greater bias in the unadjusted model. The estimates for the unadjusted and the time adjusted CSP methods provide similar results in instances when the longitudinal effect on survival was small. The standard errors were higher in the time adjusted models compared to the unadjusted models. The time adjusted PLR had larger standard errors and better coverage, compared to the unadjusted PLR. When the event rate was high (90 %) and the longitudinal association with survival was high (γ = 1.000) the unadjusted PLR showed the largest bias (0.342) compared to other methods. The bias, though still large, was attenuated in the time adjusted PLR method (0.255). The unadjusted CSP showed a bias of 0.082 compared to the time adjusted CSP bias of 0.002. For lower event rates (10 %) the bias was attenuated. The unadjusted PLR showed a bias of 0.091 and the time adjusted PLR had a bias of 0.027. The unadjusted CSP also showed a bias of 0.067 compared to the time adjusted CSP bias of 0.003. The PLR method also had larger standard errors compared to the Cox model in all simulation scenarios. In models with low event rates the standard errors for all methods were larger, as expected. The results suggest the time adjusted time dependent Cox regression methods performed best at estimating the association parameter compared to the unadjusted methods. The estimates were similar among the methods in instances when the longitudinal effect is weaker. In the supplement we also present the results for the comparison of the longitudinal effect on survival on survival with a sample size of 100 (Additional file 1: Figure S1).

**Table 4:** Comparison of longitudinal effect on survival (*N* = 1000)

Scenarios		CSP_UNADJUSTED					CSP_ADJUSTED & TDCM				
Event rate	*γ*	Estimate	SE	CP	Bias	MSE	Estimate	SE	CP	Bias	MSE
90 %	0.000	0.003	0.054	0.957	0.003	0.006	0.003	0.055	0.954	0.003	0.006
	0.500	0.498	0.055	0.954	−0.002	0.006	0.498	0.056	0.952	−0.002	0.006
	1.000	1.083	0.058	0.720	0.082	0.014	1.002	0.059	0.958	0.002	0.007
50 %	0.000	−0.001	0.075	0.953	−0.001	0.011	−0.002	0.076	0.944	−0.002	0.012
	0.500	0.499	0.070	0.947	−0.001	0.010	0.499	0.071	0.944	−0.001	0.010
	1.000	1.001	0.073	0.946	0.001	0.011	1.002	0.074	0.948	0.002	0.011
10 %	0.000	0.007	0.168	0.944	0.007	0.058	0.007	0.171	0.938	0.007	0.060
	0.500	0.501	0.158	0.947	0.001	0.051	0.501	0.161	0.946	0.001	0.053
	1.000	1.067	0.145	0.906	0.067	0.048	1.003	0.147	0.937	0.003	0.045
		PLR_UNADJUSTED					PLR_ADJUSTED				
Event rate	*γ*	Estimate	SE	CP	Bias	MSE	Estimate	SE	CP	Bias	MSE
90 %	0.000	0.003	0.066	0.957	0.003	0.008	0.003	0.067	0.957	0.003	0.009
	0.500	0.599	0.069	0.709	0.099	0.019	0.601	0.070	0.711	0.101	0.020
	1.000	1.342	0.080	0.005	0.342	0.130	1.255	0.082	0.111	0.255	0.078
50 %	0.000	−0.001	0.080	0.950	−0.001	0.013	−0.002	0.081	0.945	−0.002	0.013
	0.500	0.545	0.077	0.909	0.045	0.014	0.545	0.079	0.912	0.045	0.014
	1.000	1.109	0.084	0.746	0.109	0.026	1.109	0.086	0.744	0.109	0.027
10 %	0.000	0.007	0.170	0.945	0.007	0.059	0.007	0.173	0.937	0.007	0.061
	0.500	0.510	0.161	0.947	0.010	0.053	0.509	0.164	0.941	0.009	0.055
	1.000	1.091	0.150	0.888	0.091	0.055	1.027	0.152	0.926	0.027	0.049

*Abbreviations*: *SE* Standard Error, *CP* 95 % Coverage Probability, *MSE* Mean Square Error, *CSP_UN* Unadjusted Cross Sectional Pooling, *CSP_AD* Adjusted Cross Sectional Pooling; *PLR_UN* Unadjusted Pooled Logistic Regression, *PLR_AD* Adjusted Pooled Logistic Regression, *TDCM* Time Dependent Cox Regression Modeling

We assessed the performance of these methods in estimating the effects of Age (*α*_1_ = 0.050) and Sex (*α*_2_ = − 0.500). The standard errors were smaller in the unadjusted models compared to the time adjusted models. The results showed that the time adjusted PLR had a positive bias in the Age effect with reduced bias when the event rate is low. The PLR methods showed a negative bias in the Sex effect compared to the other methods. The Cox models yielded reliable estimates for the Sex effect in all scenarios considered. The PLR had higher estimates for the Sex effect compared to the other methods. When the event rate was 50 %, the Age effect was similar in both the time adjusted and the unadjusted models, but with extreme event rates (10 % or 90 %) there was significant bias in the unadjusted. These results suggest that the time adjusted Cox models provide more reliable estimates compared to the unadjusted Cox and logistic models. In the supplement we present results for the comparison of the longitudinal effect on survival and the Age effect on survival with a sample size of 100 (Additional file 1: Table S1). We saw similar patterns in the results compared to a sample size of 1000 (Additional file 1: Table S2).

## Application to Framingham Heart Study (FHS)

We illustrate these methods by applying them to FHS data in which lipid measurements and myocardial infarction (MI) data were collected over a period of 26 years. Since 1948 three generations of participants have been followed over the years: the Original cohort (recruited in 1948), their Offspring (recruited in 1971) and a Third Generation (recruited in 2002). Among the Offspring participants, triglycerides (TG) were measured at fairly similar time intervals of ~4 year each over a period of 26 years. The time to myocardial infarction was recorded for each participant, although some subjects were censored by the end of the study period in 2005. We log transformed the TG measures in our analysis to reduce skewness. A total of 2262 subjects with complete data were followed from 1979–2005 and data were collected at the start of each exam (Table 5). The FHS data showed a low cumulative event rate (3.71 %) for the 26-year period. In the FHS data we did see a steady increase in the TG measures from Exam 1 through Exam 6 as shown in Table 5. The mean change in TG between exams was ~ 11 mg/dL (2.40 on Natural Log Scale) with a standard deviation of ~90 mg/dL (4.50 on Natural Log Scale). So we do not expect large fluctuations in change in the Log TG measures between the exams. A total of 177 deaths were reported (7.82 %) in FHS data. Among these deaths 35 (1.55 %) were recorded prior to cardiovascular disease. Future work considering death as competing risk or event-free composite endpoints is essential in this area. In our data generation scheme and FHS data analysis we assumed missing at random (MAR) for participants who dropped out of the study with missing triglycerides at a particular exam and were censored. Additional work taking into consideration the missing data mechanism is worthy of further research.

**Table 5:** Framingham heart study data (*N* = 2262)

Characteristics	Exam 1	Exam 2	Exam 3	Exam 4	Exam 5	Exam 6
Sample size (N*)	2262	2211	2173	2118	2056	1995
Years of measurement	1979–1983	1983–1987	1987–1991	1991–1995	1995–1998	1998–2001
Age	43.32 (9.58)	47.69 (9.60)	51.15 (9.60)	54.80 (9.60)	58.87 (9.54)	61.78 (9.45)
Triglycerides	100.49 (88.77)	118.80 (123.59)	124.15 (110.18)	154.47 (133.08)	153.08 (114.92)	158.70 (112.49)
Survival time (years)	4.33 (0.60)	3.43 (0.46)	3.61 (0.46)	4.01 (0.60)	2.87 (0.86)	6.00 (1.62)
Cumulative event rate (%)	0.44 %	0.88 %	1.46 %	2.08 %	2.39 %	3.71 %
Overall event rate (%)	3.71 %					
Sex (% female)	51.19 %					

Using the methods described in section 2 we characterize the association between the longitudinal measures and time-to-event response. We use log TG at each exam for the longitudinal part of the model assuming a linear trend over time and survival time measured from exam 1 to MI or loss to follow up. We adjust for Sex and Age in all the models. The survival distribution among subjects with the events was fairly uniform and the distribution of censored subjects was skewed with most censoring occurring at the right tail end of the distribution. In Table 6 we present the estimates for Age, Sex and the association parameters. Using a 0.05 level of significance, Age, Sex and the Log of the triglyceride measures were significantly associated with the time-to-myocardial infarction in the FHS Cohort. The association parameter describes the strength of the relationship between triglycerides and MI survival; γ is the log hazard ratio for a one unit increase in the longitudinal component in the survival model. The association estimates were similar across the different methods. The Age effect estimates and standard errors were similar among the methods. The estimates ranged from 0.048–0.056 with the unadjusted analyses yielding lower estimates compared to the time adjusted analysis. The Sex effect was also consistent among the different methods.

**Table 6:** Modeling longitudinal and survival data (framingham heart study)

	AGE (*α*_1_)			SEX (*α*_2_)			LogTG (*γ*)		
Methods	Estimate	SE	P	Estimate	SE	P	Estimate	SE	P
CSP Unadjusted	0.0528	0.0103	<.0001	−1.0305	0.2434	<.0001	0.6068	0.1732	0.0005
CSP Adjusted & TDCM	0.0561	0.0119	<.0001	−1.0254	0.2435	<.0001	0.6182	0.1741	0.0004
PLR Unadjusted	0.0480	0.0101	<.0001	−1.0179	0.2444	<.0001	0.6023	0.1754	0.0006
PLR Adjusted	0.0520	0.0119	<.0001	−1.0154	0.2444	<.0001	0.6107	0.1755	0.0005

*Abbreviations*: *CSP_UN* Unadjusted Cross Sectional Pooling, *CSP_AD* Adjusted Cross Sectional Pooling, *PLR_UN* Unadjusted Pooled Logistic Regression, *PLR_AD* Adjusted Pooled Logistic Regression, *TDCM* Time Dependent Cox Regression Modeling

These FHS results are comparable to the simulation results with low event rate (10 %) and moderate association of the longitudinal measures to survival (γ = 0.500), as shown in Fig. 1. In this scenario, the association estimates were similar among the different methods.

## Discussion

In this paper we explored time dependent Cox regression methods that link longitudinal and survival data in order to quantify the association between a longitudinal process and a survival outcome, and have shown that statistical performance may be improved in models that explicitly include time as a covariate. We considered models that adjust and do not adjust for time in the Pooled Logistic Regression and the Cross Sectional Pooling methods. We conducted a series of simulations to compare these methods in their ability to estimate the link parameter. The performance was assessed through bias, coverage probabilities and Type I error rates. We analyzed data from FHS in which triglyceride measurements and Myocardial Infarction (MI) data were collected over a period of 26 years (1979–2005). To our knowledge this is the first paper that compares time adjusted and unadjusted models for modeling time dependent covariate data.

From the simulations we see that time adjusted, time dependent Cox regression methods performed best at estimating the association parameter compared to the unadjusted Cox and logistic models. Our results indicate that in some instances the PLR provides higher biased estimates and standard errors compared to the Cox models. The PLR and the time dependent Cox regression methods provide similar results when the event is rare, consistent with the results presented by Green and Symons [10]. In the unadjusted models the Age effect was attenuated depending on the association of the longitudinal measures on survival. D’Agostino et al. [6] indicated that the analyst must consider the nature of variables such as Age, which may be highly correlated with the follow-up time. There are a number of recent epidemiologic studies that implement the PLR model. Some recent work include Miguel-Yanes et al. [19]; Meigs et al. [20]; Fox et al. [21]; Ficociello et al. [22]., Marshall et al. [23]., Solomon et al. [24]. Recent studies that have also implemented the CSP approach include Schnabel et al. [25]., Magnani et al. [26]., Rienstra et al. [27]., D'Agostino [28]. The TDCM and stratified Cox model are more routine in statistical analysis. The implementation of time adjustment in PLR models is essential to obtain reliable estimates.

The FHS sample provided results that were consistent with the simulation results for a low event rate and moderate association. Thus, we did not find large differences in the estimates from the different approaches. Had the event rates been high and there were a strong association between the longitudinal measures and the survival time, we would have expected to see greater differences in the estimates from the unadjusted models, with the time-unadjusted PLR and CSP approaches having higher estimates.

There is extensive literature on comparison of the logistic regression model and the proportional hazard model. Efron [15] discusses the use of the logistic model for survival data and shows that the odds ratio estimates are approximately the same as hazard ratios. Green and Symons [10] conducted research on the conditions under which results from the Logistic regression and proportional hazards model in prospective epidemiologic studies approximate one another. They concluded that in instances where the follow-up period is short and the disease is generally rare, the regression coefficients of the logistic model approximate those of the proportional hazards model with a constant underlying hazard rate. They also stated that under the same conditions the regression coefficients have similar estimated standard errors. They provided a mathematical relationship between the Cox and the logistic models. D‘Agostino et al. [6] showed that the pooled logistic regression (PLR) is close to the time dependent covariate model. They also provided numerical examples showing the closeness of this relationship using the Framingham Heart Study. The goal of our paper was to compare these methods using simulation studies considering models that adjust and do not adjust for time in PLR and CSP and also illustrate instances where these methods differ.

In time dependent covariate models attention has to be placed on the type of covariates (internal vs. external) being considered. Kalbfleisch & Prentice [12] distinguish between external and internal covariates, where an external covariate does not require direct observation of the individual. Examples are the age of an individual and level of air pollution as a risk factor for asthma attacks. Internal covariates are generated within the individuals under study and are known only when the individual remains in the study as event-free and is uncensored. As noted by Kalbfleisch and Prentice, internal covariate processes can be affected by treatment assignment in clinical trials, or by baseline factors in observational studies such as the Framingham Heart Study. In such instances care must be exercised in the interpretation, as treatment or baseline covariate effects may be reflected predominantly in the time-varying covariate process [12]. We acknowledge the potential limitations of predicting survival with internal time-varying covariates given the conceptualization of the conditional survival function; however, we used internal time-dependent covariates in our Framingham Heart Study example given the clinical interest in the relationship between TG values and risk of myocardial infarction. Thus care needs to be taken in the interpretation of the results given the use of an internal time-varying factor that may also be an intermediate variable between the baseline factors and the outcome.

One limitation to our study is that we did not consider measurement errors or missing data issues that may arise from longitudinal covariate data that are potentially missing at failure times. In many longitudinal studies participants may drop out early from the study which may lead to missing data in both the failure times as well as the time dependent covariates. Such issues can be addressed in a mixed effects model in which a random effect can be used to capture the individual specific longitudinal trajectories with missing data. Complete-case methods, which discard incomplete observations in survival regression models, may potentially lead to inefficient or biased estimates. In the presence of missing data, there are a number of likelihood and imputation methods for addressing missing data given the observed data. In our study we did not address these issues as our simulations and examples are based on no missing data at each time point. Models that consider measurement error may be more representative of the underlying process. In our study, using Framingham Heart Data, individuals were censored at time of death. A drawback to this approach is that death without prior myocardial infarction may be considered a competing event to our outcome. The main objective of our study was to present an overview of these methods for modeling time dependent covariates in the context of longitudinal and survival data. Exploring methods that consider death as competing risk or event-free composite endpoints are worthy of further research. A limitation to every simulation study is that the results are dependent on the scenarios examined. In this paper, we evaluated a range of models to provide broader insight, but our conclusions must be limited to the scenarios that we examined. In the simulation models the assumption of proportional hazards was considered in all scenarios. Further work is needed to better understand the circumstances when the PLR may differ from Cox models in non-proportional hazard models.

## Conclusions

In this study we compare three methods for quantifying the association between a longitudinal process and a survival outcome. We characterize the relation between the longitudinal measures and time-to-event in models that account for the time at which the longitudinal measures are recorded. In general, we recommend the use of a stratified Cox model with time intervals or a TDCM when the time-to-event is available, both of which account for time. When the time of the response is not available, a PLR approach may be applied adjusting for the Time interval at which the time dependent covariate measures were taken. If event rates are high and the association between longitudinal measures and survival are strong, the PLR approach without adjustment for time is not recommended. The Cox model provides greater use of the available data compared to the PLR by including time. Thus, when time is available, we recommend using the TDCM or equivalently the stratified CSP approaches with time intervals. Survival analyses that explicitly account for the times at which time dependent covariates are measured appear to provide more reliable estimates compared to unadjusted analyses.

## Additional file

Additional file 1: Figure S1. Estimates and Confidence Intervals for Association Parameter (*N* = 100). Values are presented as estimates and 95 % confidence intervals for the link parameter. Varying link parameter (0.00, 0.50, and 1.00); varying event rates (10 %, 50 %, and 90 %). Abbreviations: CSP_UN: Unadjusted Cross Sectional Pooling; CSP_AD: Adjusted Cross Sectional Pooling; PLR_UN: Unadjusted Pooled Logistic Regression; PLR_AD: Adjusted Pooled Logistic Regression; TDCM: Time Dependent Cox Regression Modeling. (DOCX 84 kb)

## Abbreviations

CSP: Cross sectional pooling
CSP_AD: Adjusted Cross-sectional pooling
CSP_UN: Unadjusted Cross-sectional pooling
FHS: Framingham Heart Study
MI: Myocardial Infarction
MSE: Mean square error
PLR: Pooled Logistic Regression
PLR_AD: Adjusted Pooled logistic regression
PLR_UN: Unadjusted Pooled logistic regression
TDCM: Time dependent covariate modeling
TG: Triglycerides
