# Supplementary material for: A comparison of time dependent Cox regression, pooled logistic regression and cross sectional pooling with simulations and an application to the Framingham Heart Study
Source: BMC Med Res Methodol. 2016 Nov 3;16:148. doi: 10.1186/s12874-016-0248-6 (PMC5094095; doi:10.1186/s12874-016-0248-6)
Supplement: Additional file 1: Figure S1. — Estimates and Confidence Intervals for Association Parameter (N = 100). Values are presented as estimates and 95 % confidence intervals for the link parameter. Varying link parameter (0.00, 0.50, and 1.00); varying event rates (10 %, 50 %, and 90 %). Abbreviations: CSP_UN: Unadjusted Cross Sectional Pooling; CSP_AD: Adjusted Cross Sectional Pooling; PLR_UN: Unadjusted Pooled Logistic Regression; PLR_AD: Adjusted Pooled Logistic Regression; TDCM: Time Dependent Cox Regression Modeling. (DOCX 84 kb) [file 12874_2016_248_MOESM1_ESM.docx]

**SUPPLEMENTAL TABLES AND FIGURES**

**A Comparison of Time Dependent Cox Regression, Pooled Logistic Regression and Cross Sectional Pooling with Simulations and an Application to the Framingham Heart Study**

**List of Figures and Tables**

| **Comparing Longitudinal and Age Effect on Survival** | | |
| --- | --- | --- |
| Figure S1 | Comparison of Longitudinal Effect on Survival (N = 100, Link = γ) | 1 |
| Table S1 | Comparison of Age Effect on Survival (N = 100, $\alpha_{1}$ = 0.050) | 2 |
| Table S2 | Comparison of Age Effect on Survival (N = 1000, $\alpha_{1}$ = 0.050) | 3 |


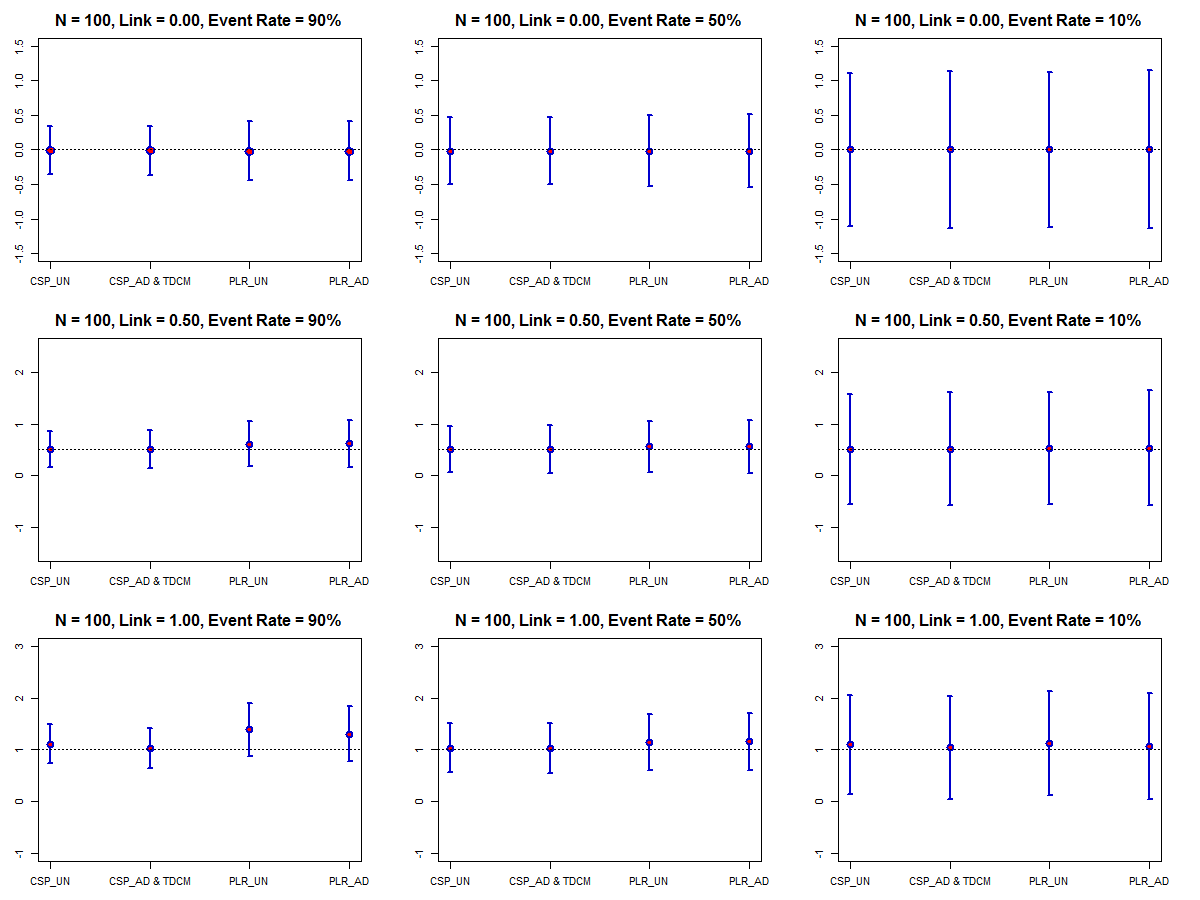


| **Table S1: Comparison of Age Effect on Survival (N = 100,** $\boldsymbol{\alpha}_{\boldsymbol{1}}$**= 0.050)** | | | | | | | | | | | |
| --- | --- | --- | --- | --- | --- | --- | --- | --- | --- | --- | --- |
| **Scenarios** | | **CSP_UNADJUSTED** | | | | | **CSP_ADJUSTED & TDCM** | | | | |
| **Event Rate** | $\boldsymbol{\gamma}$ | **Estimate (**$\alpha_{1}\boldsymbol{)}$ | **SE** | **CP** | **Bias** | **MSE** | **Estimate (**$\alpha_{1}\boldsymbol{)}$ | **SE** | **CP** | **Bias** | **MSE** |
| 90% | 0.000 | 0.053 | 0.014 | 0.937 | 0.003 | 0.000 | 0.052 | 0.024 | 0.944 | 0.002 | 0.001 |
|  | 0.500 | 0.053 | 0.014 | 0.950 | 0.003 | 0.000 | 0.052 | 0.024 | 0.931 | 0.002 | 0.001 |
|  | 1.000 | 0.011 | 0.014 | 0.185 | -0.039 | 0.002 | 0.052 | 0.025 | 0.930 | 0.002 | 0.001 |
| 50% | 0.000 | 0.052 | 0.017 | 0.950 | 0.002 | 0.001 | 0.052 | 0.033 | 0.948 | 0.002 | 0.002 |
|  | 0.500 | 0.052 | 0.016 | 0.946 | 0.002 | 0.001 | 0.055 | 0.030 | 0.940 | 0.005 | 0.002 |
|  | 1.000 | 0.051 | 0.016 | 0.964 | 0.001 | 0.001 | 0.050 | 0.031 | 0.957 | 0.000 | 0.002 |
| 10% | 0.000 | 0.053 | 0.040 | 0.956 | 0.003 | 0.004 | 0.052 | 0.075 | 0.958 | 0.002 | 0.012 |
|  | 0.500 | 0.055 | 0.039 | 0.957 | 0.005 | 0.003 | 0.054 | 0.072 | 0.942 | 0.004 | 0.011 |
|  | 1.000 | 0.012 | 0.033 | 0.771 | -0.038 | 0.004 | 0.051 | 0.065 | 0.952 | 0.001 | 0.009 |
|  | | | | | | | | | | | |
|  | | **PLR_UNADJUSTED** | | | | | **PLR_ADJUSTED** | | | | |
| **Event Rate** | $\boldsymbol{\gamma}$ | **Estimate (**$\alpha_{1}\boldsymbol{)}$ | **SE** | **CP** | **Bias** | **MSE** | **Estimate (**$\alpha_{1}\boldsymbol{)}$ | **SE** | **CP** | **Bias** | **MSE** |
| 90% | 0.000 | 0.064 | 0.017 | 0.877 | 0.014 | 0.001 | 0.063 | 0.029 | 0.926 | 0.013 | 0.002 |
|  | 0.500 | 0.064 | 0.017 | 0.886 | 0.014 | 0.001 | 0.063 | 0.030 | 0.919 | 0.013 | 0.002 |
|  | 1.000 | 0.014 | 0.018 | 0.453 | -0.036 | 0.002 | 0.065 | 0.032 | 0.926 | 0.015 | 0.002 |
| 50% | 0.000 | 0.056 | 0.019 | 0.945 | 0.006 | 0.001 | 0.056 | 0.035 | 0.951 | 0.005 | 0.003 |
|  | 0.500 | 0.057 | 0.018 | 0.940 | 0.007 | 0.001 | 0.061 | 0.034 | 0.935 | 0.011 | 0.002 |
|  | 1.000 | 0.056 | 0.018 | 0.950 | 0.006 | 0.001 | 0.055 | 0.035 | 0.962 | 0.005 | 0.002 |
| 10% | 0.000 | 0.054 | 0.041 | 0.956 | 0.004 | 0.004 | 0.054 | 0.077 | 0.958 | 0.004 | 0.013 |
|  | 0.500 | 0.056 | 0.040 | 0.957 | 0.006 | 0.003 | 0.056 | 0.073 | 0.940 | 0.006 | 0.012 |
|  | 1.000 | 0.012 | 0.034 | 0.781 | -0.038 | 0.004 | 0.053 | 0.067 | 0.951 | 0.003 | 0.009 |
| **Abbreviations**: SE: Standard Error; CP: 95% Coverage Probability; MSE: Mean Square Error; CSP_UN: Unadjusted Cross Sectional Pooling; CSP_AD: Adjusted Cross Sectional Pooling; PLR_UN: Unadjusted Pooled Logistic Regression; PLR_AD: Adjusted Pooled Logistic Regression; TDCM: Time Dependent Cox Regression Modeling | | | | | | | | | | | |

| **Table S2: Comparison of Age Effect on Survival (N = 1000,** $\boldsymbol{\alpha}_{\boldsymbol{1}}$**= 0.050)** | | | | | | | | | | | |
| --- | --- | --- | --- | --- | --- | --- | --- | --- | --- | --- | --- |
| **Scenarios** | | **CSP_UNADJUSTED** | | | | | **CSP_ADJUSTED & TDCM** | | | | |
| **Event Rate** | $\boldsymbol{\gamma}$ | **Estimate (**$\boldsymbol{\alpha}_{\mathbf{1}}\mathbf{)}$ | **SE** | **CP** | **Bias** | **MSE** | **Estimate (**$\alpha_{1}\boldsymbol{)}$ | **SE** | **CP** | **Bias** | **MSE** |
| 90% | 0.000 | 0.050 | 0.004 | 0.941 | 0.000 | 0.000 | 0.050 | 0.007 | 0.937 | 0.000 | 0.000 |
|  | 0.500 | 0.050 | 0.004 | 0.956 | 0.000 | 0.000 | 0.050 | 0.007 | 0.957 | 0.000 | 0.000 |
|  | 1.000 | 0.010 | 0.004 | 0.000 | -0.040 | 0.002 | 0.050 | 0.008 | 0.939 | 0.000 | 0.000 |
| 50% | 0.000 | 0.050 | 0.005 | 0.949 | 0.000 | 0.000 | 0.051 | 0.010 | 0.961 | 0.000 | 0.000 |
|  | 0.500 | 0.050 | 0.005 | 0.955 | 0.000 | 0.000 | 0.050 | 0.009 | 0.957 | 0.000 | 0.000 |
|  | 1.000 | 0.051 | 0.005 | 0.949 | 0.001 | 0.000 | 0.050 | 0.009 | 0.949 | 0.000 | 0.000 |
| 90% | 0.000 | 0.049 | 0.012 | 0.950 | -0.001 | 0.000 | 0.049 | 0.022 | 0.960 | -0.001 | 0.001 |
|  | 0.500 | 0.050 | 0.012 | 0.959 | 0.000 | 0.000 | 0.050 | 0.021 | 0.947 | 0.000 | 0.001 |
|  | 1.000 | 0.011 | 0.010 | 0.027 | -0.039 | 0.002 | 0.051 | 0.019 | 0.946 | 0.001 | 0.001 |
|  | | | | | | | | | | | |
|  | | **PLR_UNADJUSTED** | | | | | **PLR_ADJUSTED** | | | | |
| **Event Rate** | $\boldsymbol{\gamma}$ | **Estimate (**$\alpha_{1}\boldsymbol{)}$ | **SE** | **CP** | **Bias** | **MSE** | **Estimate (**$\alpha_{1}\boldsymbol{)}$ | **SE** | **CP** | **Bias** | **MSE** |
| 90% | 0.000 | 0.061 | 0.005 | 0.475 | 0.011 | 0.000 | 0.061 | 0.009 | 0.796 | 0.011 | 0.000 |
|  | 0.500 | 0.061 | 0.005 | 0.488 | 0.011 | 0.000 | 0.060 | 0.009 | 0.805 | 0.010 | 0.000 |
|  | 1.000 | 0.012 | 0.005 | 0.000 | -0.038 | 0.002 | 0.062 | 0.010 | 0.759 | 0.012 | 0.000 |
| 50% | 0.000 | 0.054 | 0.006 | 0.906 | 0.004 | 0.000 | 0.054 | 0.011 | 0.938 | 0.004 | 0.000 |
|  | 0.500 | 0.055 | 0.006 | 0.875 | 0.005 | 0.000 | 0.054 | 0.010 | 0.937 | 0.004 | 0.000 |
|  | 1.000 | 0.056 | 0.006 | 0.832 | 0.006 | 0.000 | 0.055 | 0.011 | 0.924 | 0.005 | 0.000 |
| 10% | 0.000 | 0.050 | 0.012 | 0.950 | 0.000 | 0.000 | 0.050 | 0.023 | 0.959 | 0.000 | 0.001 |
|  | 0.500 | 0.051 | 0.012 | 0.957 | 0.001 | 0.000 | 0.051 | 0.021 | 0.943 | 0.001 | 0.001 |
|  | 1.000 | 0.011 | 0.010 | 0.034 | -0.039 | 0.002 | 0.052 | 0.020 | 0.947 | 0.002 | 0.001 |
| **Abbreviations**: SE: Standard Error; CP: 95% Coverage Probability; MSE: Mean Square Error; CSP_UN: Unadjusted Cross Sectional Pooling; CSP_AD: Adjusted Cross Sectional Pooling; PLR_UN: Unadjusted Pooled Logistic Regression; PLR_AD: Adjusted Pooled Logistic Regression; TDCM: Time Dependent Cox Regression Modeling | | | | | | | | | | | |
